# Potential JAK2 Inhibitors from Selected Natural Compounds: A Promising Approach for Complementary Therapy in Cancer Patients

**DOI:** 10.1155/2024/1114928

**Published:** 2024-04-26

**Authors:** Samaneh Vaziri-Amjad, Reza Rahgosha, Amir Taherkhani

**Affiliations:** ^1^Department of Oral and Maxillofacial Medicine, School of Dentistry, Hamadan University of Medical Sciences, Hamadan, Iran; ^2^Research Center for Molecular Medicine, Hamadan University of Medical Sciences, Hamadan, Iran

## Abstract

**Background:**

Janus-activated kinase 2 (JAK2) plays a pivotal role in numerous essential biological processes, including proliferation, apoptosis, and metastasis in human cells. Prior studies have indicated that inhibiting JAK2 could be a promising strategy to mitigate cell proliferation and induce apoptosis in tumor cells.

**Objectives:**

This study aimed to estimate the binding affinity of 79 herbal compounds, comprising 46 flavonoids, 21 anthraquinones, and 12 cinnamic acids, to the ATP-binding cleft of JAK2 to identify potential herbal inhibitors of JAK2.

**Methods:**

The binding affinities between ligands and JAK2 were calculated utilizing AutoDock 4.0 software in conjunction with the Cygwin environment. Cross-validation was conducted using the Schrödinger tool. Molecular dynamics simulations were employed to evaluate the stability of docked poses for the most significant JAK2 inhibitors. Furthermore, the Discovery Studio Visualizer tool was utilized to elucidate interactions between the top-ranked JAK2 inhibitors and residues within the JAK2 ATP-binding site.

**Results:**

Twelve flavonoids, two anthraquinones, and three cinnamic acids demonstrated substantial binding affinities to the protein kinase domain of the receptor, with a criterion of Δ*G*_binding_ < −10 kcal/mol. Among the studied flavonoids, anthraquinones, and cinnamic acid derivatives, orientin, chlorogenic acid, and pulmatin emerged as the most potent JAK2 inhibitors, exhibiting Δ*G*_binding_ scores of −14.49, −11.87, and −10.76 kcal/mol, respectively. Furthermore, the docked poses of orientin, pulmatin, and chlorogenic acid remained stable throughout 60 ns computer simulations. The average root mean square deviation values calculated for JAK2 when complexed with orientin, chlorogenic acid, and pulmatin were 2.04 Å, 2.06 Å, and 1.95 Å, respectively.

**Conclusion:**

This study underscores the robust inhibitory potential of orientin, pulmatin, and chlorogenic acid against JAK2. The findings hold promise for the development of novel and effective drugs for cancer treatment.

## 1. Introduction

**Figure 1:**
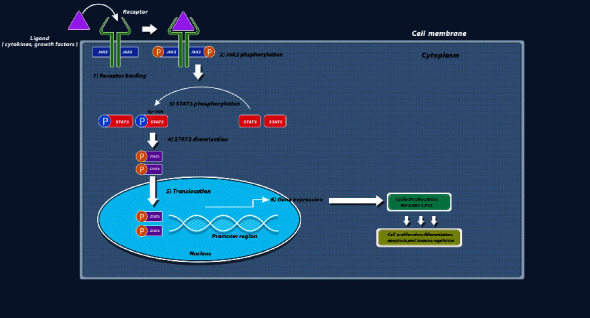
An illustrative depiction portraying the operational mechanism of JAK2 across various pathways linked to the initiation and progression of cancer. JAK2, janus-activated kinase 2.

The Janus-activated kinase (JAK) family includes JAK1, JAK2, JAK3, and tyrosine kinase 2 (TYK2) [1]. This enzyme family is involved in many vital biological processes associated with cell differentiation, proliferation, metastasis, and apoptosis in human organs [2–5]. JAKs are upstream regulators of signal transducer and activator of transcription 3 (STAT3) [6], which is a well-known oncogenic factor in several human cancers [7]. Accumulating evidence indicates that JAK2 phosphorylates STAT3, leading to the upregulation of several factors, including B-cell lymphoma 2 (Bcl-2) [8], proto-oncogene c-Myc [9], cyclin D1, and vascular endothelial growth factor (VEGF) [10], which are associated with cell survival and proliferation. Further, the upregulation of the JAK2/STAT3 signaling pathway leads to the increased expression of intercellular adhesion molecule 1 (ICAM1) and isovalerate--CoA ligase CCL2 (CCL2). ICAM1 mediates the metastasis of breast cancer cells into the brain. Besides, CCL2 plays a critical role in attracting macrophages to cancer cells [11]. The oncogenic role of JAK2 in other malignancies, including colorectal [12], non-small cell lung [13], ovarian [5], gastric [14], pancreatic [15], prostate [16], and renal [17] cancers, has also been demonstrated. Oh et al. [18] showed that a herbal compound named licochalcone C inhibited the JAK2 activity, leading to downregulating JAK2/STAT3 signaling and reducing proliferation and enhancing apoptosis in oral squamous cell carcinoma cells. Therefore, a positive correlation has been demonstrated between enhanced expression and/or activity of JAK2 and tumorigenesis in many cancers [19]. Moreover, the critical impact of JAK2 in inflammation, aging, and hematopoiesis has been reviewed by Perner et al. [20]. Several reports have claimed that JAK2 inhibition might be a promising approach to diminishing cell proliferation and triggering apoptosis in cancer cells [21].  Figure 1 illustrates the intricate mechanism through which JAK2 influences diverse roles within distinct biological pathways, culminating in the process of tumorigenesis.

**Figure 2:**
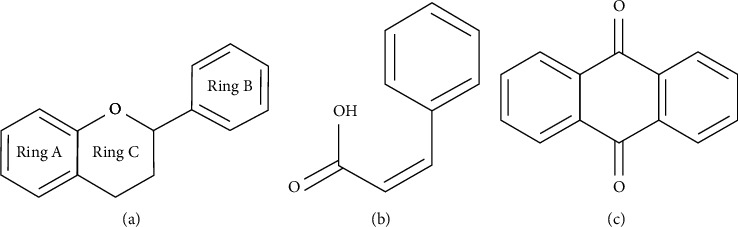
Lead scaffolds of (a) flavonoids, (b) cinnamic acids, and (c) anthraquinones.

It has been reported that natural compounds could affect JAK/STAT3 signaling, leading to enhanced apoptosis and reduced cell proliferation in cancer cells [22]. Flavonoids, cinnamic acids, and anthraquinones are three major classes of herbal compounds in medicinal plants [23–25]. Polyphenolic compounds known as flavonoids, which have a basic C_6_-C_3_-C_6_ structure (Figure 2(a)) [26], are abundant in foods like green tea and chocolate [27]. Apples [28, 29], plums, cherries [28, 30], berries [31, 32], olives [33], onions [34], beans [35], spinach [36], and shallot [37] are also rich sources of flavonoids. Flavonoids have demonstrated anticancer effects by downregulating the cell cycle process, cell proliferation, and invasiveness behavior of tumor cells. Besides, these compounds trigger the apoptosis process in cancer cells [27]. Cinnamic acids are aromatic carboxylic acid compounds with the basic chemical structure of C_6_–C_3_ (Figure 2(b)) [38]. These metabolites have attracted the attention of scientists because of their structural diversity, widespread distribution, and low toxicity with several beneficial properties, including antioxidant [39], anti-inflammatory [40], anticancer [41], antidepressant [42], hypoglycemic [43], and neuroprotective [44] effects. Anthraquinones are a class of compounds derived from the 9,10-anthracenedione backbone, with various functional groups like hydroxyl, methyl, and carboxyl attached at different positions (Figure 2(c)) [45]. They have also exerted anti-inflammatory [46], antihyperlipidemic [47], immunoregulation [48], and antitumor characteristics in previous reports. They are mainly extracted from *Rubiaceae*, *Leguminosae*, *Polygonaceae*, *Scrophulariaceae*, *Verbenaceae*, *Rhamnaceae*, *Valerianaceae*, and *Liliaceae* [24].

Molecular docking and dynamics simulations have emerged as vital tools for rapidly discovering and optimizing novel drugs against several diseases including COVID-19 [49, 50]. Sureja et al. [51] screened twenty-seven antiviral lignan derivatives for inhibiting the emerging pathogen SARS-CoV-2. Molecular docking predicted interactions between viral enzymes and lignans. The emerging compounds clemastatin B and erythro-strebluslignanol G exhibited strong binding to several viral proteins. Further dynamics simulations supported these emerging inhibitors' potential, particularly clemastatin B and savinin. Furthermore, Shah et al. [52] evaluated 30 emerging antiviral phytoconstituents against three coronavirus proteins using docking to assess binding affinity, alongside pharmacokinetics, toxicity, and bioactivity profiling. Five emerging compounds showed favorable profiles, with biscoclaurine and norreticuline demonstrating high affinity to key viral proteins, indicating possible efficacy in curbing the emerging virus' replication.

Targeting upregulated genes in cancer has been considered to treat patients effectively [53]. Herein, it was hypothesized that JAK2 might be a potential target of flavonoids, cinnamic acids, and anthraquinones, leading to antiproliferative effects in cancer cells. Therefore, a computational drug discovery method was designed to assess the binding affinity of selected herbal isolates, including flavonoids, cinnamic acids, and anthraquinones mainly found in commonly used fruits and vegetables to the JAK2 ATP-binding site [18, 54]. Afterward, top-ranked herbal compounds in each class were selected, and the resistance of their binding sites was examined using computational molecular dynamics (MD) in 60 nanoseconds (ns). Interactions between the most potent JAK2 inhibitors and residues within the protein's ATP-binding cleft were also studied. The results were compared with two positive control inhibitors of JAK2. The findings might lead to the identification of novel anticancer drugs based on herbal metabolites.

## 2. Materials and Methods

### 2.1. Preparing Structures of JAK2 and Herbal Metabolites

To examine the binding affinity between herbal isolates and the human JAK2 ATP-binding site, a docking methodology was executed using AutoDock 4.0. The JAK2 three-dimensional (3D) structure was obtained from the Research Collaboration for Structural Bioinformatics (RCSB) database [55] (Protein Data Bank [PDB] entry, 2B7A; X-ray resolution, 2 Å) [18, 54].

The decision to center this research around 2B7A resulted from a meticulous evaluation of multiple factors, including structural analysis and an extensive review of relevant literature. Initially, scrutiny was directed towards the JAK2 entry within the Uniprot database, specifically emphasizing the protein's three-dimensional structure determined via X-ray crystallography. Among the ten available PDB IDs, those with resolutions exceeding 2 Å (2W1I, 2XA4, 3FUP, 3IO7, 3IOK, and 3JY9) were disregarded in favor of higher-quality structures. Consequently, four options remained under consideration, including 2B7A, 3E62, 3E63, and 3E64. Subsequently, the PubMed database was consulted to ascertain which structures had been employed for prior molecular docking analyses of JAK2 inhibition. It was discovered that Oh et al. [18] utilized 2B7A to assess the binding interactions of Licochalcone C, highlighting its apoptotic effects in cancer cells through the regulation of the JAK/STAT signaling pathway. Drawing from this established precedent in the literature, 2B7A was ultimately chosen as the focal point for this research.

The 2B7A file involved two polypeptide chains labeled A and B, from which chain A, with 290 residues, was selected by the Notepad++ tool for further in silico analyses. The JAK2 inhibitor, 2-TERT-BUTYL-9-FLUORO-3,6-DIHYDRO-7H-BENZ[H]-IMIDAZ[4,5-F] ISOQUINOLINE-7-ONE (PDB entry, 1ZA; PubChem entry, 5494425) was removed from the PDB file, and subsequently, energy minimizing (EM) was applied to chain A using Swiss-pdbViewer version 4.1.0 to reach the most stable structure of the enzyme. Critical residues inside the JAK2 ATP-binding cleft were identified by reviewing the previous studies by Lucet et al. [54] and Oh et al. [18] and analyzing the interactions between 1ZA and residues within the JAK2 kinase domain using BIOVIA Discovery Studio Visualizer (DSV) version 19.1.0.18287.

Seventy-nine herbal compounds, including 46 flavonoids, 21 anthraquinones, and 12 cinnamic acids, were selected to dock with the JAK2 protein tyrosine kinase (PTK) domain. Two ligands were also assigned as positive control inhibitors of the enzyme; these ligands included the standard drug named ruxolitinib (DrugBank entry, DB08877 [56]; PubChem entry, 25126798) and 1ZA (PubChem entry, 5494425). The structures of the ligands were initially downloaded as SDF formats and subsequently converted into PDB files. All ligands were energy minimized, followed by our previous reports [38, 45, 57].

### 2.2. Virtual Screening Analysis

*In silico* analyses were executed using a Windows-based computer with the following characteristics: System type, 64-bit; Installed RAM, 64 GB DDR5; Processor, Intel 24-Core i9-13900KF. Using the AutoDock 4.0 tool [58, 59], the Kollmann charge and polar hydrogen bonds were added to the receptors' structure, and local electric charge and rotational motion were included in the ligands' structures. Fourteen residues including Leu855, Val863, Ala880, Val911, Met929, Glu930, Tyr931, Leu932, Pro933, Asp939, Arg980, Leu983, Glu993, and Asp994 were indicated within the JAK2 kinase domain. Accordingly, the grid box was set to X-dimension, 54; Y-dimension, 76; Z-dimension, 66; X-center, 115.868 Å; Y-center, 65.728 Å; Z-center, 8.298 Å, and spacing, 0.375 Å to cover all atoms within the ATP-binding site. The receptor and ligands were saved as PDBQT files, and the Lamarckian Genetic Algorithm method was used to predict the position of the compounds inside the receptor. After that, the Cygwin environment [60], available at https://www.cygwin.com/, was used for docking simulations. All components were docked 50 times with the JAK2 ATP-binding cleft, and subsequently, the results were grouped according to the root mean square deviation (RMSD) of 2.0 Å. The most negative Δ*G*_binding_ score of the largest cluster in the RMSD table was assigned as the binding affinity between the ligands and JAK2.

### 2.3. Cross-Validation Study

Molecular docking studies were conducted to cross-validate the strongest predicted JAK2 inhibitor from flavonoids, cinnamic acid derivatives, and anthraquinones. Compounds were initially selected based on favorable binding free energies calculated with AutoDock. Further validation was achieved utilizing Schrödinger Maestro (v10.2) [61, 62] and the Glide docking algorithm to predict ligand binding affinities. The prime MM-GBSA calculations were performed to determine relative binding energies for the ligands under investigation. This orthogonal docking approach allows confirmation of the top-predicted JAK2 inhibitor [63].

### 2.4. MD Simulations and Interaction Mode Analysis

Top-ranked inhibitors from flavonoids, cinnamic acid derivatives, anthraquinones, and JAK2 control inhibitors, were selected for further MD analyses in 60 ns computer simulations; this was performed using the Discovery Studio Client software version 16.1.0.15350. MD simulations were executed with the following advanced settings: solvent, water; target temperature, 310 K; force field, CHARMm; solvation model, explicit periodic boundary; minimum distance from the boundary, 10 Å; charge distribution, point; cell shape, orthorhombic [57]. The RMSD of the JAK2 backbone atoms (PDB entry, 2B7A) and the root mean square fluctuation (RMSF) of the JAK2 residues were analyzed during 60 ns MD simulations. Moreover, the DSV software defined hydrogen bonds and hydrophobic interactions between top-ranked herbal compounds, the most potent positive control compound, and the JAK2 ATP-binding site.

To ensure the robustness of the MD simulations, particularly focusing on the JAK2 backbone atoms throughout a 60-nanosecond trajectory, a series of validation measures were implemented. Initially, an equilibration phase was executed preceding the production MD run, during which the system reached a stable state by applying positional restraints. Subsequently, gradual heating of the system to the target temperature of 310 Kelvin was carried out, followed by a gradual pressure equilibration process. The selection of the CHARMM force field [64], renowned for its accuracy in capturing biomolecular behavior, was meticulously undertaken to ensure compatibility with both ligand and JAK2 protein parameters. Utilizing 1ZA as a standard JAK2 inhibitor served as a reference for the dynamics of JAK2-1ZA within our simulations, thereby enabling comparative analyses with the behavior of JAK2 complexed with the top-ranked flavonoid, anthraquinone, and cinnamic acid.

### 2.5. Pharmacokinetics, Toxicity, and Bioavailability of Top-Ranked Compounds

The pharmacokinetic properties of the highest-performing herbal isolates across various classes were predicted using the SwissADME online platform (https://www.swissadme.ch/) [65], with key parameters related to absorption, distribution, metabolism, and excretion (ADME) being encompassed within this analysis. Within this ADME modeling framework, several critical pharmacokinetic characteristics for these compounds were considered, such as gastrointestinal absorption potential, blood-brain barrier permeability, possible cytochrome P450 enzyme inhibition interactions, and susceptibility as a P-glycoprotein substrate. Furthermore, the assessment of ligand carcinogenic potential as an indicator of compound toxicity [66] was conducted utilizing the toxCSM web server, accessible at https://biosig.lab.uq.edu.au/toxcsm [67].

Furthermore, the *in silico* prediction of oral bioavailability [68] for these compounds was conducted using the SwissADME web application. The likelihood of acceptable human intestinal absorption and permeability for the compound structures under investigation was predicted utilizing the Bioavailability Score model within SwissADME. Property descriptors were calculated from the molecular structure input, and a quantitative multiple linear regression prediction was made between 0 and 1, with higher scores indicating better bioavailability [65]. Compounds with high oral bioavailability, generally considered to have scores above 0.55, were identified [69]. The compound structures in SMILES format were input into the Bioavailability Score model's web interface using the default parameters, and the resultant bioavailability predictions were recorded and compared.

## 3. Results

### 3.1. The Binding Affinity of Herbal Compounds to JAK2

**Figure 3:**
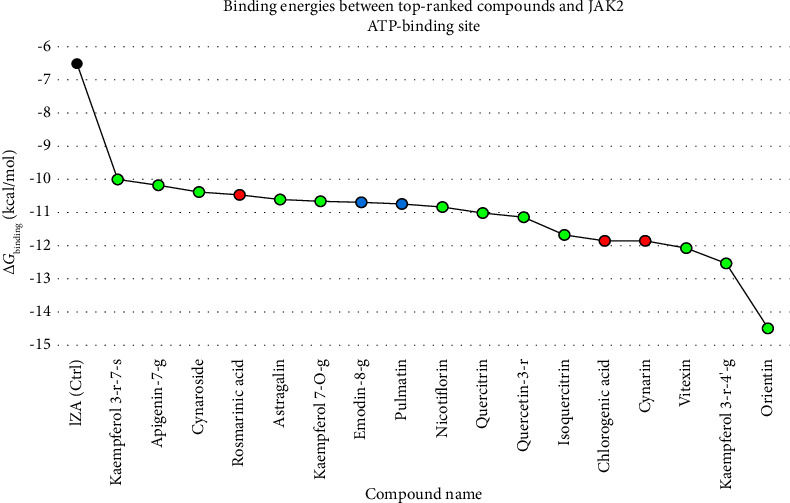
Δ*G*_binding_ values between top-ranked herbal compounds, a positive control inhibitor, and JAK2 ATP-binding site. The *X*-axis shows the compounds' names, and the *Y*-axis demonstrates the gibbs free binding energy in kcal/mol units. Green, blue, red, and black spots present flavonoids, anthraquinones, cinnamic acids, and a positive control inhibitor. JAK2, janus-activated kinase 2.

In the present study, the compounds that bonded to the JAK2 ATP-binding cleft with the criterion of Δ*G*_binding_ < −10 kcal/mol were assigned the most potent inhibitors of the enzyme. From flavonoids, the compounds included orientin, kaempferol 3-rutinoside-4′-glucoside, vitexin, isoquercitrin, quercetin-3-rhamnoside, quercitrin, nicotiflorin, kaempferol 7-O-glucoside, astragalin, cynaroside, apigenin-7-glucoside, and kaempferol 3-rutinoside-7-sophoroside. From anthraquinones, pulmatin, and emodin-8-glucoside were the most potent JAK2 inhibitors. Likewise, chlorogenic acid, cynarin, and rosmarinic acid were indicated as top-ranked JAK2 inhibitors from cinnamic acid derivatives. Among the two positive control inhibitors, 1ZA demonstrated the most binding affinity to JAK2 ATP-binding site with a binding energy of −6.55 kcal/mol, suggesting the most potent herbal inhibitors of JAK2 in this study bonded with the enzyme's ATP-binding site more robustly than the control inhibitors did (Table 1).  Table 2 showcases the energy types involved between the top-ranked herbal compounds, 1ZA, and the JAK2 ATP-binding cleft, along with the chemical structures of these top-ranked compounds.  Figure 3 compares the Gibbs free energy of binding between top-ranked herbal ligands in this study, 1ZA, and JAK2 kinase domain. The Δ*G*_binding_ value between a ligand and receptor is calculated from the following equations [70, 71]: Δ*G*_binding_ = Intermolecular Energy + TotalInternalEnergy + Torsional Free Energy − Unbound System's Energy.

(1) $$\begin{array}{c} \Delta G_{\text{binding}}=G_{\text{complex}}\;–\;\left(G_{\text{protein}}\;–\;G_{\text{ligand}}\right). \end{array}$$

### 3.2. Cross-Validations

Subsequent prime MM-GBSA calculations provided binding energy estimates, ranking chlorogenic acid as the strongest binder followed by orientin and then pulmatin (Table 3). The Schrödinger suites confirmed these three phytochemicals as notably occupying the catalytic region of JAK2 with high predicted affinity. This justified their selection for more rigorous analysis through MD simulations. Complexes derived from AutoDock were utilized as starting structures for the MD studies.

### 3.3. MD Simulation Analyses

**Figure 4:**
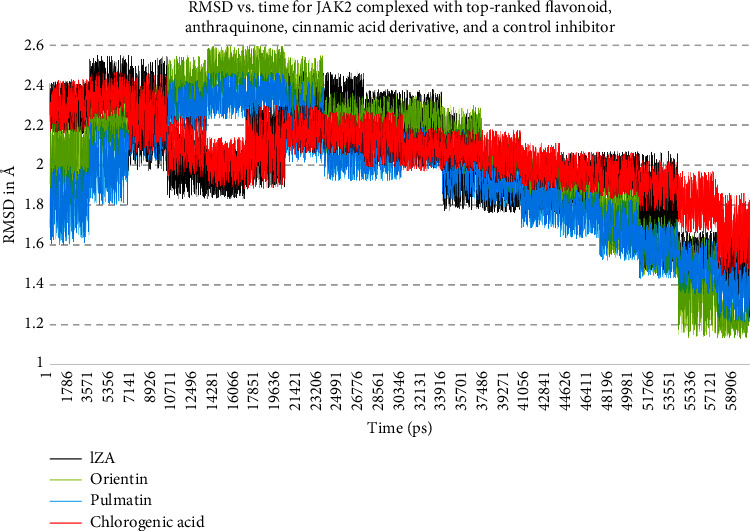
RMSD for JAK2 backbone atoms complexed with top-ranked flavonoid, anthraquinone, cinnamic acid derivative, and a positive control inhibitor. The *X*-axis presents the simulation time, and the *Y*-axis demonstrates the RMSD. JAK2, janus-activated kinase 2.; RMSD, root-mean-square deviations.

**Figure 5:**
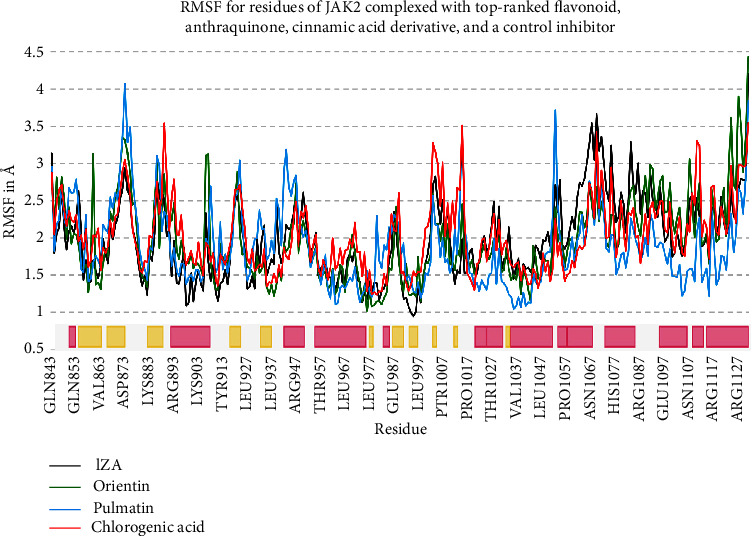
RMSF for JAK2 backbone atoms complexed with 1ZA, orientin, pulmatin, and chlorogenic acid. Pink and yellow regions demonstrate *α*-helix and *β*-strand secondary structures from the RCSB databank. The *X*-axis illustrates the name and number of residues, while the *Y*-axis shows the RMSF. JAK2, janus-activated kinase 2; RMSF, root mean square fluctuation.

**Figure 6:**
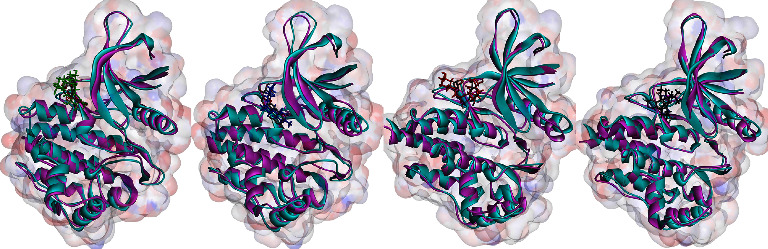
Superimposed structures of JAK2 before and after MD simulations. Cyan and purple colors display the JAK2 before and after MD simulations. Green, blue, red, and black colors present orientin, pulmatin, chlorogenic acid, and 1ZA after 60 ns MD simulations. JAK2, janus-activated kinase 2; MD, molecular dynamics.

Orientin, pulmatin, and chlorogenic acid were demonstrated to be the most potent JAK2 inhibitors from flavonoids, anthraquinones, and cinnamic acid derivatives. The Δ*G*_binding_ for orientin, pulmatin, and chlorogenic acid was calculated as −14.49, −10.76, and −11.87 k cal/mol, respectively. Besides, the most binding affinity between JAK2 and the enzyme's control inhibitors was recorded for 1ZA. Thus, MD analyses were performed in 60 ns simulations to study the strength of the docked poses of these ligands. According to the RMSD graph, it could be assumed that the RMSD of the enzyme's backbone is decreasing after ∼20 ns computer simulations. The average RMSD for JAK2 complexed with orientin, pulmatin, chlorogenic acid, and 1ZA was determined as 2.04, 1.95, 2.06, and 2.03 Å, respectively (Figure 4). In addition, the RMSF graph showed that *α*-helixes and *β*-sheets were more resistant secondary structures than irregular secondary structures and *β*-turns (Figure 5).  Figure 6 demonstrates the superimposed structures of JAK2 complexed with orientin, pulmatin, chlorogenic acid, and 1ZA before and after MD simulations.

### 3.4. Interaction Modes

**Figure 7:**
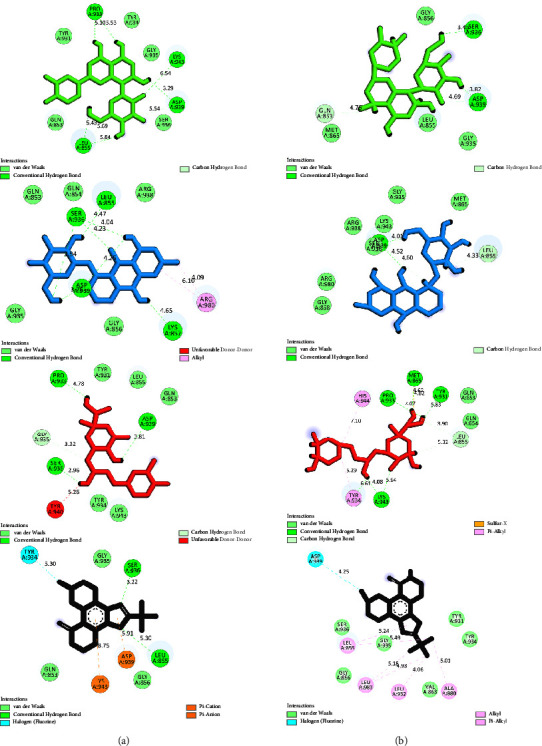
Interaction types between orientin (green), pulmatin (blue), chlorogenic acid (red), 1ZA (black), and JAK2 ATP-binding site. The (a and b) images show interactions before and after MD simulation, respectively. JAK2, janus-activated kinase 2; MD, molecular dynamics.

Interaction types between the most potent JAK2 inhibitors in this study, 1ZA, and residues inside the enzyme's ATP-binding site were uncovered using the DSV tool. For orientin, pulmatin, chlorogenic acid, and 1ZA, interactions were also studied after 60 ns MD simulations (Table 4 and Figure 7). Isoquercitrin and pulmatin formed the greatest number of hydrogen interactions with JAK2 from flavonoids and anthraquinones; these metabolites demonstrated six and seven H-bonds with the residues within the JAK2 ATP-binding cleft, respectively. Chlorogenic acid and cynarin showed the most H-bonds (*n* = 4) with the residues incorporated in the JAK2 kinase domain. Orientin exhibited one H-bond before the MD simulation, while this flavonoid formed three hydrogen interactions with JAK2 residues after the MD simulation. Furthermore, pulmatin and chlorogenic acid demonstrated four hydrogen bonds after MD simulations.

### 3.5. ADME, Toxicity (ADMET), and Bioavailability Predictions

As outlined in Table 5, the foremost herbal compounds, when scrutinized for carcinogenesis prediction, exhibited notable safety profiles. Nonetheless, the examination of ADME and bioavailability unveiled distinct attributes among the compounds under investigation. Specifically, all assessed compounds displayed minimal gastrointestinal absorbance, with none permeating the blood-brain barrier. Moreover, predictive analyses suggested that kaempferol 3-rutinoside-4′-glucoside, quercetin-3-rhamnoside, quercitrin, cynaroside, apigenin-7-glucoside, pulmatin, emodin-8-glucoside, and cynarin could potentially induce drug resistance by inhibiting g-proteins. Furthermore, none of the compounds exhibited inhibition of cytochrome P450, indicating favorable metabolic pathways within the human system. Notably, among flavonoids, vitexin and apigenin-7-glucoside displayed commendable bioavailability, achieving a score of 0.55. Similarly, the anthraquinones pulmatin and emodin-8-glucoside demonstrated favorable bioavailability attributes, also scoring 0.55. Among cinnamic acid derivatives, rosmarinic acid emerged with the highest bioavailability score of 0.56, surpassing all other compounds assessed.

## 4. Discussion

The JAK2/STAT3 pathway is one of the most critical signal transduction pathways mediating cell differentiation and proliferation. The JAK2/STAT3 hyperactivity results in the onset and development of cancer [72]. Herein, a computational virtual screening was executed to identify potential JAK2 inhibitors from 79 herbal metabolites, including 46 flavonoids, 21 anthraquinones, and 12 cinnamic acid derivatives. According to the present results, orientin, pulmatin, and chlorogenic acid revealed the most binding affinities to the JAK2 ATP-binding site among flavonoids, anthraquinones, and cinnamic acid derivatives.

### 4.1. Pharmacodynamics and Primary Sources of Leading Compounds

The Δ*G*_binding_ and *K*i values between orienting and JAK2 kinase domain were calculated as −14.49 kcal/mol and 23.81 pM. Orientin demonstrated one H-bond with Asp939 within the JAK2 ATP-binding site before MD simulation. Besides, orientin exhibited three H-bonds with Gln853, Ser936, and Asp939 after MD simulation, suggesting that the interaction between Asp939 and orientin was stable after 60 ns MD simulation.

Orientin is predominantly present in several medicinal plants, such as *Patrinia villosa Juss*, *Lindsaeaceae*, *Phyllostachys*, *Trollius chinensis Bunge*, and *Indocalalamus latifolius* [73]. Its notable anticancer properties include the inhibition of cell proliferation, induction of apoptosis, and suppression of tumor invasion and metastasis. These effects are attributed to its regulation of key molecular pathways and gene expression. Orientin has demonstrated therapeutic promise as an anticancer agent, as evidenced by its effectiveness against various cancer cell lines, including esophageal and breast cancer cells [74–76].

The Δ*G*_binding_ and *K*i values between pulmatin (chrysophanol 8-glucoside) and JAK2 ATP-binding site were recorded as −10.76 kcal/mol and 13.07 nM. Before MD simulation, pulmatin formed two hydrophobic interactions and seven H-bonds with Leu855, Lys857, Ser936, Asp939, and Arg980 inside the JAK2 protein kinase domain. After MD simulation, pulmatin demonstrated four H-bonds with Leu855 and Asp939, suggesting that the interactions between Leu855 and Asp939 and pulmatin were stable after 60 ns computer simulation.

The discovery of chrysophanol originated from *Rheum rhabarbarum*, an herbaceous perennial plant categorized within the *Polygonaceae* family [77]. Chrysophanol has demonstrated potent anti-inflammatory and anti-proliferative properties based on the *in vitro* evidence provided by the analyzed studies. The compound not only inhibited cell growth and pro-inflammatory cytokine production but also modulated key regulatory proteins like p53, NF-*κ*B, and caspase-3. These findings suggest that chrysophanol could be a promising candidate for the development of treatments for conditions characterized by excessive inflammation and cell proliferation [78, 79].

The Δ*G*_binding_ and *K*i values between chlorogenic acid and JAK2 ATP-binding cleft were estimated as −11.87 kcal/mol and 1.98 nM, respectively. Chlorogenic acid exhibited four H-bonds with Pro933, Gly935, Ser936, and Asp939 corporated inside the JAK2 ATP-binding cleft before MD simulation. Also, chlorogenic acid formed two hydrophobic interactions and four H-bonds with Leu855, Met865, Pro933, Tyr934, Lys943, and His944, suggesting that the H-bond between Pro933 and chlorogenic acid was stable after 60 ns MD simulation.

Coffee is a main source of chlorogenic acid [80]. According to previous reports, chlorogenic acid demonstrated significant medicinal properties, particularly in the context of cancer prevention and therapy. The evidence underlines chlorogenic acid's ability to induce apoptosis and cell cycle arrest, as well as modulating signaling pathways involved in cancer cell survival [81–83]. Therefore, a high degree of confidence that chlorogenic acid possesses potent anticancer activities and may serve as a promising candidate for adjunctive cancer therapy.

### 4.2. Comparative Review with Existing Literature

Yuan et al. [84] demonstrated that eriocitrin (a lemon flavanone) suppressed the phosphorylation of STAT3 in MCF-7 cells by inhibiting the JAK2, leading to the enhanced apoptosis process. Furthermore, ericocitrin activated proapoptotic factors Bax, caspase 7, 8, and 9 and downregulated Bcl-2 and Bcl-x.

Li et al. [85] reported that an amide anthraquinone derivative named 1-nitro-2-acyl anthraquinone glycine (C10) had a high binding affinity to JAK2, leading to downregulation of the JAK2/STAT3 signaling pathway, resulting in the induced cell cycle arrest and inhibiting cell proliferation in HCT116 and HT29 colon cancer cells.

Kim et al. [86] demonstrated that pretreatment of RAW264.7 cells with chlorogenic acid inhibited phosphorylation of JAK2 and STAT3, leading to the suppression of lipopolysaccharide-induced nitric oxide, interleukin 1*β* (IL-1*β*), IL-6, tumor necrosis factor-*α*, and MMP-2.

### 4.3. Structure-Activity Relationships (SARs)

In the context of Δ*G*_binding_, the following structure-activity relationships (SARs) are suggested:

#### 4.3.1. Flavonoids

The binding affinity of flavonoids to the JAK2 active site is augmented by glycosylation at various positions. This includes sugar conjugation to carbon 3 of ring C (as exemplified by isoquercitrin, quercetin-3-rhamnoside, quercitrin, nicotiflorin, and astragalin), glycosidic bonds forming at carbon 7 of ring A (as seen in kaempferol 7-O-glucoside, cynaroside, and apigenin-7-glucoside), and carbon 8 of ring A (as observed in orientin and vitexin).

#### 4.3.2. Cinnamic Acids

Among cinnamic acid derivatives, compounds featuring two C6 aromatic rings (such as cynarin and rosmarinic acid), or a single benzene ring linked to a sugar-like moiety, exhibit heightened binding affinity to JAK2. Particularly noteworthy is chlorogenic acid, characterized by a distinctive structure comprising a phenolic acid core, quinic acid component, and an ester linkage. Although not strictly categorized as a sugar, the presence of the polyhydroxylated cyclic carboxylate quinic acid imparts structural characteristics reminiscent of glycosylation. The inclusion of this moiety in chlorogenic acid may similarly contribute to favorable interactions within the JAK2 active site.

#### 4.3.3. Anthraquinones

Glycosylation at the R5 position on ring A of the anthraquinone core scaffold enhances the binding affinity for JAK2, as demonstrated by pulmatin and emodin-8-glucoside.

### 4.4. Anticancer Properties of Himalayan Plants

Tariq et al. [87] conducted an extensive examination of Himalayan medicinal plants with potential anticancer properties, encompassing ethnopharmacological, phytochemical, and pharmacological aspects. Their study identified 64 such plants, primarily from India, highlighting the rich diversity of anticancer flora in the region. This research by Tariq et al. [87] is significant for its thorough documentation of Himalayan plant species with potential therapeutic implications against cancer.

In a study by Gupta et al. [88], the focus was on the diosgenin-enriched extract derived from Paris polyphylla rhizome, a plant traditionally employed in the Indian Himalayas for wound healing and anticancer purposes. The authors quantified the diosgenin content in the extract and conducted assessments of its *in vitro* antioxidant, *in vivo* anti-inflammatory, and *in vitro* anticancer activities. Their findings revealed notable cytotoxicity against various cancer cell lines, particularly breast cancer cells, implicating diosgenin as the principal phytochemical responsible for these effects.

Manhas et al. [89] reported the isolation of a rare bisaryl anthraquinone antibiotic, named Setomimycin, from a novel Streptomyces strain discovered in the Shivalik region of the NW Himalayas. This compound demonstrated *in vitro* anticancer and anti-migratory effects, evidenced by reduced expression of MEK and ERK pathways in cancer cell lines. Furthermore, *in vivo* studies exhibited a substantial reduction in tumor weight and volume in an orthotopic mouse mammary carcinoma model.

## 5. Conclusion

This study calculated the binding affinities of 79 plant-based metabolites to the ATP-binding site of JAK2. Twelve flavonoids, two anthraquinones, and three cinnamic acid derivatives exhibited strong predicted binding, with Δ*G*_binding_ values under −10 kcal/mol. The flavonoid orientin, along with the cinnamic acid derivative chlorogenic acid and the anthraquinone pulmatin, showed the highest binding affinities among the compounds tested at −14.49 kcal/mol, −11.87 kcal/mol, and −10.76 kcal/mol, respectively. MD simulations over 60 ns indicated stable binding for these three lead compounds within the JAK2 site, with average RMSD values of 2.04 Å (orientin), 2.06 Å (chlorogenic acid), and 1.95 Å (pulmatin) relative to the initially docked conformations. Our results suggest that orientin, pulmatin, and chlorogenic acid may act as potent JAK2 inhibitors. These compounds could lead to novel therapeutics for cancer, pending confirmation via *in vitro* and *in vivo* experiments. Overall, this study predicts novel scaffold families with activity against JAK2 as a starting point for further drug development efforts targeting this clinically important kinase.

## Figures and Tables

**Table 1:** Gibbs free energy of binding and inhibition constant values between the tested flavonoids, anthraquinones, cinnamic acids, control inhibitors, and JAK2 ATP-binding cleft.

PubChem entry	Ligand name	Δ*G*_binding_	Inhibition constant
*(A) Flavonoids*			
5281675	Orientin	−14.49	23.81 pM
44258844	Kaempferol 3-rutinoside-4′-glucoside	−12.54	643.71 pM
5280441	Vitexin	−12.07	1.42 nM
5280804	Isoquercitrin	−11.69	2.68 nM
5353915	Quercetin-3-rhamnoside	−11.16	6.64 nM
5280459	Quercitrin	−11.03	8.29 nM
5318767	Nicotiflorin	−10.86	10.88 nM
10095180	Kaempferol 7-O-glucoside	−10.68	14.89 nM
5282102	Astragalin	−10.62	16.45 nM
5280637	Cynaroside	−10.40	23.77 nM
5280704	Apigenin-7-glucoside	−10.19	33.67 nM
44258853	Kaempferol 3-rutinoside-7-sophoroside	−10.02	44.96 nM
5280805	Rutin	−9.97	49.29 nM
72936	Sophoraflavanone G	−9.79	66.64 nM
5281600	Amentoflavone	−9.66	83.58 nM
471	Dihydroquercetin	−9.55	99.31 nM
5280544	Herbacetin	−9.45	117.75 nM
5280343	Quercetin	−9.16	191.44 nM
9911508	Astragarin	−9.05	232.52 nM
442664	Vicenin-2	−8.95	273.18 nM
439533	Taxifolin	−8.81	348.58 nM
5281672	Myricetin	−8.70	419.29 nM
5281654	Isorhamnetin	−8.51	577.45 nM
5316673	Afzelin	−8.41	681.83 nM
5281614	Fisetin	−8.35	752.61 nM
5281612	Diosmetin	−8.33	783.91 nM
5281670	Morin	−8.15	1.05 *μ*M
5280445	Luteolin	−8.07	1.22 *μ*M
5280681	3-O-Methylquercetin	−7.85	1.77 *μ*M
25201019	Ponciretin	−7.82	1.86 *μ*M
5280863	Kaempferol	−7.80	1.93 *μ*M
5317435	Fustin	−7.75	2.97 *μ*M
638278	Isoliquiritigenin	−7.63	2.55 *μ*M
124052	Glabridin	−7.49	3.24 *μ*M
5281607	Chrysin	−7.48	3.30 *μ*M
1203	Epicatechin	−7.39	3.82 *μ*M
5280443	Apigenin	−7.39	7.12 *μ*M
72281	Hesperetin	−7.38	3.90 *μ*M
639665	XanthohuMol	−7.30	4.44 *μ*M
5318998	Licochalcone A	−7.13	5.96 *μ*M
9064	Catechin	−7.10	6.23 *μ*M
14309735	Xanthogalenol	−6.92	8.51 *μ*M
443639	Epiafzelechin	−6.81	10.11 *μ*M
629440	Hemileiocarpin	−6.71	12.06 *μ*M
10680	Flavone	−5.86	50.71 *μ*M
5280378	Formononetin	−5.73	63.22 *μ*M

*(B) Anthraquinones*			
442731	Pulmatin (chrysophanol-8-0-glucoside)	−10.76	13.07 nM
99649	Emodin-8-glucoside	−10.71	14.02 nM
126456371	Aloe emodin 8-glucoside	−8.89	305.84 nM
6683	Purpurin	−8.48	549.18 nM
3220	Emodin	−8.43	659.07 nM
10207	Aloe-emodin	−8.17	1.02 *μ*M
10208	Chrysophanol	−8.05	1.26 *μ*M
10168	Rhein	−8.04	1.28 *μ*M
3663	Hypericin	−7.97	1.43 *μ*M
361510	Emodic acid	−7.88	1.68 *μ*M
10459879	Sennidin B	−7.86	1.74 *μ*M
10639	Physcion	−7.79	1.94 *μ*M
2950	Danthron	−7.77	2.02 *μ*M
6293	Alizarin	−7.68	2.35 *μ*M
101286218	Rhodoptilometrin	−7.49	3.25 *μ*M
3083575	Obtusifolin	−6.58	15.00 *μ*M
160712	Nordamnacanthal	−6.36	21.69 *μ*M
92826	Sennidin A	−6.22	27.43 *μ*M
124062	Rubiadin	−6.15	31.02 *μ*M
442753	Knipholone	−6.11	146.75 *μ*M
2948	Damnacanthal	−5.67	70.16 *μ*M

*(C) Cinnamic acids*			
1794427	Chlorogenic acid	−11.87	1.98 nM
6124212	Cynarin	−11.87	1.99 nM
5281792	Rosmarinic acid	−10.48	20.66 nM
5281759	Caffeic acid 3-glucoside	−8.06	1.24 *μ*M
5281787	Caffeic acid phenethyl ester	−7.84	1.99 *μ*M
637540	o-Coumaric acid	−6.29	24.45 *μ*M
5372945	N-p-coumaroyltyramine	−6.28	24.94 *μ*M
637775	Sinapinic acid	−5.65	72.61 *μ*M
689043	Caffeic acid	−5.63	75.13 *μ*M
445858	Ferulic acid	−5.23	145.82 *μ*M
444539	Cinnamic acid	−4.97	228.75 *μ*M
637542	p-Coumaric acid	−4.88	264.73 *μ*M

*(D) Control inhibitors*			
25126798	Ruxolitinib	−6.33	23.01 *μ*M
5494425	1ZA	−6.55	15.89 *μ*M

JAK2, janus-activated kinase 2; 1ZA, 2-TERT-BUTYL-9-FLUORO-3,6-DIHYDRO-7H-BENZ[H]-IMIDAZ[4,5-F] ISOQUINOLINE-7-ONE; pM, picomolar; nM, nanomolar; *μ*M, micromolar.

**Table 2:** The chemical structures of the top herbal inhibitors identified in this study were compared to the structure of a known JAK2 inhibitor used as a positive control.

Ligand name	Intermolecular energy (kcal/mol)	Total internal energy (kcal/mol)	Torisonal free energy (kcal/mol)	Unbound System's energy (kcal/mol)	Free binding energy (kcal/mol)	Chemical structure
*(A) Flavonoids*						
Orientin	−8.30	−10.68	+3.58	−0.91	−14.49	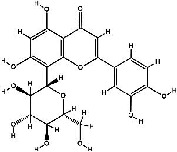
Kaempferol 3-rutinoside-4′-glucoside	−4.90	−16.69	+5.97	−3.08	−12.54	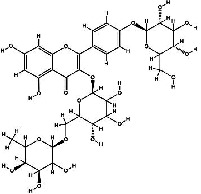
Vitexin	−8.74	−7.09	+3.28	−0.49	−12.07	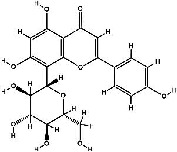
Isoquercitrin	−8.87	−8.93	+3.88	−2.23	−11.69	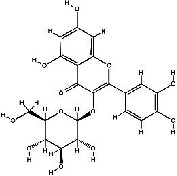
Quercetin-3-rhamnoside	−7.52	−9.35	+3.28	−2.43	−11.16	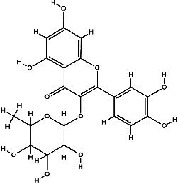
Quercitrin	−8.79	−7.68	+3.28	−2.16	−11.03	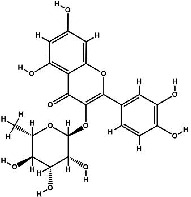
Nicotiflorin	−7.72	−10.78	+4.77	−2.87	−10.86	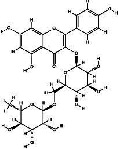
Kaempferol 7-O-glucoside	−7.89	−6.81	+2.68	−1.34	−10.68	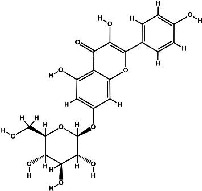
Astragalin	−7.52	−7.40	+2.68	−1.62	−10.62	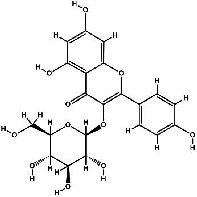
Cynaroside	−7.21	−7.55	+2.68	−1.68	−10.40	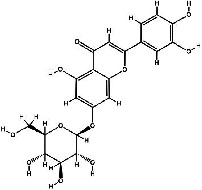
Apigenin-7-glucoside	−9.16	−5.63	+3.28	−1.32	−10.19	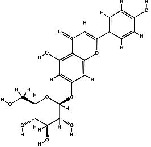

*(B) Anthraquinones*						
Pulmatin	−9.00	−6.23	+2.98	−1.50	−10.76	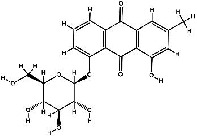
Emodin-8-glucoside	−9.32	−6.15	+3.28	−1.47	−10.71	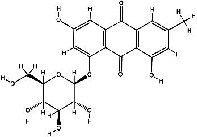

*(C) Cinnamic acid derivatives*						
Chlorogenic acid	−6.61	−10.96	+4.18	−1.53	−11.87	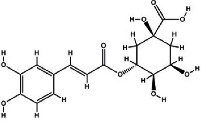
Cynarin	−6.26	−13.89	+6.26	−2.02	−11.87	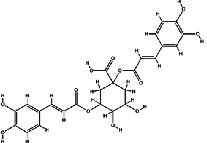
Rosmarinic acid	−8.33	−7.66	+4.47	−1.03	−10.48	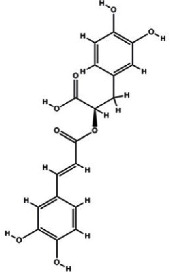

*(D) Control inhibitor*						
1ZA	−6.84	−0.28	+0.30	−0.28	−6.55	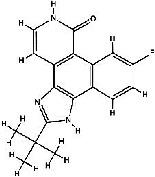

JAK2, janus-activated kinase 2; 1ZA, 2-TERT-BUTYL-9-FLUORO-3,6-DIHYDRO-7H-BENZ[H]-IMIDAZ[4,5-F] ISOQUINOLINE-7-ONE; pM, picomolar; nM, nanomolar; *μ*M, micromolar. The interaction energies between these compound structures and the enzyme's active site were also analyzed.

**Table 3:** Schrödinger maestro relative binding-free energies, obtained by prime MM-GBSA (in kcal/mol), of the top-ranked flavonoid, cinnamic acid, and anthraquinone compounds based on the AutoDock tool against the JAK2 active site (PDB ID: 2B7A).

Compound name	MM-GBSA-dG binding energy
Orientin	−21.28
Chlorogenic acid	−24.13
Pulmatin	−14.76

JAK2, janus-activated kinase 2.

**Table 4:** Interactions modes between JAK2 catalytic site, top-ranked herbal inhibitors in this study, and JAK2 positive control inhibitor.

Ligand name	Hydrogen bond (distance A)	Hydrophobic interaction (distance A)
*(A) Flavonoids*		
Orientin (before MD)	ASP939 (3.29)	NA
Orientin (after MD)	Ser936 (3.45); Asp939 (3.82); Gln853 (4.76)	NA
Kaempferol 3-rutinoside-4′-glucoside	ASP939 (4.82); GLN854 (4.06);	LYS857 (4.92)
Vitexin	ASP939 (4.70); LYS943 (3.85);	NA
Isoquercitrin	LEU855 (4.83); SER936 (3.29, 3.33, 2.71); ASP939 (4.09, 3,12)	HIS944 (6.11)
Quercetin-3-rhamnoside	LEU855 (4.77, 3.52)	LYS943 (4.49); HIS944 (6.70)
Quercitrin	LEU855 (3.50, 4.74)	HIS944 (6.83)
Nicotiflorin	LYS943 (4.98); SER936 (2.99)	LYS943 (4.76)
Kaempferol 7-O-glucoside	GLN853 (4.80); ASP939 (3.50); SER936 (2.71); PRO933 (4.81);	HIS944 (6.28); LYS943 (5.24)
Astragalin	TYR934 (4.87); LEU855 (4.86, 4.51)	HIS944 (6.35); LYS943 (4.53); LEU855 (5.96); IZA2001 (8.24)
Cynaroside	GLN853 (4.81); LYS943 (4.68); TYR934 (4.85, 3.86);	NA
Apigenin-7-glucoside	LEU855 (3.12); ASP939 (4.04); SER936 (2.99); TYR934 (4.17)	NA

*(B) Anthraquinones*		
Pulmatin (before MD)	SER936 (2.94, 4.23, 4.04); LEU855 (4.47); ASP939 (3.75, 4.25); LYS857 (4.65)	ARG980 (4.09, 6.62)
Pulmatin (after MD)	LEU855 (4.33); ASP939 (4.01, 4.52, 4.60)	NA
Emodin-8-glucoside	ASP939 (3.80)	TYR934 (4.36); TYR940 (5.20); HIS944 (5.39, 6.58)

*(C) Cinnamic acids*		
Chlorogenic acid (before MD)	SER936 (2.96); GLY935 (3.32); ASP939 (3.81); PRO933 (4.78)	NA
Chlorogenic acid (after MD)	PRO933 (4.07); MET865 (4.62); LEU855 (3.9); LYS943 (4.08)	HIS944 (7.1); TYR934 (5.29)
Cynarin	TYR931 (3.88, 4.50, 4.24); GLU877 (3.93);	PRO933 (4.84); VAL878 (4.75)
Rosmarinic acid	ASP939 (3.60, 4.28, 3.64)	NA

*(D) Control inhibitor*		
1ZA (before MD)	SER936 (3.22)	LEU855 (5.28); IZA2001 (7.29, 6.44, 5.02)
1ZA (after MD)	NA	ALA880 (5.01); LEU932 (4.06); LEU983 (4.93, 5.18); LEU855 (5.24, 5.49)

JAK2, janus-activated kinase 2; 1ZA, 2-TERT-BUTYL-9-FLUORO-3,6-DIHYDRO-7H-BENZ[H]-IMIDAZ[4,5-F] ISOQUINOLINE-7-ONE; pM, picomolar; nM, nanomolar; *μ*M, micromolar.

**Table 5:** Predicted ADMET for top-ranked herbal metabolites in this study.

Ligand name	GI abs	BBB permeant	P-gp substrate	CYP1A2 inhibitor	CYP2C19 inhibitor	CYP2C9 inhibitor	CYP2D6 inhibitor	CYP3A4 inhibitor	Carcinogenesis	Bioavailability score
Orientin	Low	No	No	No	No	No	No	No	High safety	0.17
Kaempferol 3-rutinoside-4′-glucoside	Low	No	Yes	No	No	No	No	No	High safety	0.17
Vitexin	Low	No	No	No	No	No	No	No	High safety	0.55
Isoquercitrin	Low	No	No	No	No	No	No	No	High safety	0.17
Quercetin-3-rhamnoside	Low	No	Yes	No	No	No	No	No	High safety	0.17
Quercitrin	Low	No	Yes	No	No	No	No	No	High safety	0.17
Nicotiflorin	Low	No	No	No	No	No	No	No	High safety	0.17
Kaempferol 7-O-glucoside	Low	No	No	No	No	No	No	No	High safety	0.17
Astragalin	Low	No	No	No	No	No	No	No	High safety	0.17
Cynaroside	Low	No	Yes	No	No	No	No	No	High safety	0.17
Apigenin-7-glucoside	Low	No	Yes	No	No	No	No	No	High safety	0.55
Pulmatin	Low	No	Yes	No	No	No	No	No	High safety	0.55
Emodin-8-glucoside	Low	No	Yes	No	No	No	No	No	High safety	0.55
Chlorogenic acid	Low	No	No	No	No	No	No	No	High safety	0.11
Cynarin	Low	No	Yes	No	No	No	No	No	High safety	0.11
Rosmarinic acid	Low	No	No	No	No	No	No	No	High safety	0.56

GI, gastrointestinal; abs, absorption; BBB, blood–brain barrier; P-gp, p-glycoprotein; CYP, cytochrome p-450.

## Data Availability

The datasets used and/or analyzed during the current study are available from the corresponding author upon reasonable request.

## References

[1] Aaronson D. S., Horvath C. M. (2002). A road map for those who don’t know JAK-STAT. *Science*.

[2] Rawlings J. S., Rosler K. M., Harrison D. A. (2004). The JAK/STAT signaling pathway. *Journal of Cell Science*.

[3] Igaz P., Toth S., Falus A. (2001). Biological and clinical significance of the JAK-STAT pathway; lessons from knockout mice. *Inflammation Research*.

[4] O’Shea J. J., Gadina M., Schreiber R. D. (2002). Cytokine signaling in 2002: new surprises in the Jak/Stat pathway. *Cell*.

[5] Kandala P. K., Srivastava S. K. (2012). Regulation of Janus-activated kinase-2 (JAK2) by diindolylmethane in ovarian cancer in vitro and in vivo. *Drug discoveries & therapeutics*.

[6] Harada D., Takigawa N., Kiura K. (2014). The role of STAT3 in non-small cell lung cancer. *Cancers*.

[7] Yu H., Lee H., Herrmann A., Buettner R., Jove R. (2014). Revisiting STAT3 signalling in cancer: new and unexpected biological functions. *Nature Reviews Cancer*.

[8] Zhao R., Follows G. A., Beer P. A. (2008). Inhibition of the Bcl-xL deamidation pathway in myeloproliferative disorders. *New England Journal of Medicine*.

[9] Catalano S., Giordano C., Rizza P. (2009). Evidence that leptin through STAT and CREB signaling enhances cyclin D1 expression and promotes human endometrial cancer proliferation. *Journal of Cellular Physiology*.

[10] Kupferman M. E., Jayakumar A., Zhou G. (2009). Therapeutic suppression of constitutive and inducible JAK\STAT activation in head and neck squamous cell carcinoma. *Journal of Experimental Therapeutics and Oncology*.

[11] Wang S., Liang K., Hu Q. (2017). JAK2-binding long noncoding RNA promotes breast cancer brain metastasis. *Journal of Clinical Investigation*.

[12] Park S. Y., Lee C. J., Choi J. H. (2019). The JAK2/STAT3/CCND2 Axis promotes colorectal Cancer stem cell persistence and radioresistance. *Journal of Experimental & Clinical Cancer Research*.

[13] Liang L., Hui K., Hu C. (2019). Autophagy inhibition potentiates the anti-angiogenic property of multikinase inhibitor anlotinib through JAK2/STAT3/VEGFA signaling in non-small cell lung cancer cells. *Journal of Experimental & Clinical Cancer Research*.

[14] Wu X., Tao P., Zhou Q. (2017). IL-6 secreted by cancer-associated fibroblasts promotes epithelial-mesenchymal transition and metastasis of gastric cancer via JAK2/STAT3 signaling pathway. *Oncotarget*.

[15] Fan X., Fu H., Xie N., Guo H., Fu T., Shan Y. (2021). Inhibition of JAK2/STAT3 signaling pathway by panaxadiol limits the progression of pancreatic cancer. *Aging*.

[16] Yu C., Fan Y., Zhang Y., Liu L., Guo G. (2022). LINC00893 inhibits the progression of prostate cancer through miR-3173-5p/SOCS3/JAK2/STAT3 pathway. *Cancer Cell International*.

[17] Fang Z., Tang Y., Fang J. (2013). Simvastatin inhibits renal cancer cell growth and metastasis via AKT/mTOR, ERK and JAK2/STAT3 pathway. *PLoS One*.

[18] Oh H. N., Seo J. H., Lee M. H. (2018). Licochalcone C induced apoptosis in human oral squamous cell carcinoma cells by regulation of the JAK2/STAT3 signaling pathway. *Journal of Cellular Biochemistry*.

[19] Qian C. J., Yao J., Si J. M. (2011). Nuclear JAK2: form and function in cancer. *The Anatomical Record*.

[20] Perner F., Perner C., Ernst T., Heidel F. H. (2019). Roles of JAK2 in aging, inflammation, hematopoiesis and malignant transformation. *Cells*.

[21] Chen K. F., Tai W. T., Huang J. W. (2011). Sorafenib derivatives induce apoptosis through inhibition of STAT3 independent of Raf. *European Journal of Medicinal Chemistry*.

[22] Shan X., Zhou X., Yang J., Wang Y., Deng Y., Zhang M. (2010). Inhibitory effect of cucurbitacin E on the proliferation of ovarian cancer cells and its mechanism. *ChinesSe Journal of Cancer*.

[23] Meshram R. J., Bagul K. T., Pawnikar S. P., Barage S. H., Kolte B. S., Gacche R. N. (2020). Known compounds and new lessons: structural and electronic basis of flavonoid-based bioactivities. *Journal of Biomolecular Structure and Dynamics*.

[24] Wang D., Wang X. H., Yu X. (2021). Pharmacokinetics of anthraquinones from medicinal plants. *Frontiers in Pharmacology*.

[25] Diniz L. R. L., Souza M. T. d S., Barboza J. N., Almeida R. N., Sousa D. P. (2019). Antidepressant potential of cinnamic acids: mechanisms of action and perspectives in drug development. *Molecules*.

[26] Taherkhani A., Orangi A., Moradkhani S., Khamverdi Z. (2021). Molecular docking analysis of flavonoid compounds with matrix metalloproteinase-8 for the identification of potential effective inhibitors. *Letters in Drug Design and Discovery*.

[27] Kopustinskiene D. M., Jakstas V., Savickas A., Bernatoniene J. (2020). Flavonoids as anticancer agents. *Nutrients*.

[28] Arts I. C., van de Putte B., Hollman P. C. (2000). Catechin contents of foods commonly consumed in The Netherlands. 1. Fruits, vegetables, staple foods, and processed foods. *Journal of Agricultural and Food Chemistry*.

[29] Vrhovsek U., Rigo A., Tonon D., Mattivi F. (2004). Quantitation of polyphenols in different apple varieties. *Journal of Agricultural and Food Chemistry*.

[30] de Pascual-Teresa S., Santos-Buelga C., Rivas-Gonzalo J. C. (2000). Quantitative analysis of flavan-3-ols in Spanish foodstuffs and beverages. *Journal of Agricultural and Food Chemistry*.

[31] Määttä-Riihinen K. R., Kamal-Eldin A., Törrönen A. R. (2004). Identification and quantification of phenolic compounds in berries of Fragaria and Rubus species (family Rosaceae). *Journal of Agricultural and Food Chemistry*.

[32] Wu X., Gu L., Prior R. L., McKay S. (2004). Characterization of anthocyanins and proanthocyanidins in some cultivars of Ribes, Aronia, and Sambucus and their antioxidant capacity. *Journal of Agricultural and Food Chemistry*.

[33] Romani A., Mulinacci N., Pinelli P., Vincieri F. F., Cimato A. (1999). Polyphenolic content in five tuscany cultivars of Olea europaea L. *Journal of Agricultural and Food Chemistry*.

[34] Slimestad R., Fossen T., Vågen I. M. (2007). Onions: a source of unique dietary flavonoids. *Journal of Agricultural and Food Chemistry*.

[35] Mejri F., Selmi S., Martins A. (2018). Broad bean (Vicia faba L.) pods: a rich source of bioactive ingredients with antimicrobial, antioxidant, enzyme inhibitory, anti-diabetic and health-promoting properties. *Food & Function*.

[36] Pandjaitan N., Howard L., Morelock T., Gil M. (2005). Antioxidant capacity and phenolic content of spinach as affected by genetics and maturation. *Journal of Agricultural and Food Chemistry*.

[37] Fattorusso E., Iorizzi M., Lanzotti V., Taglialatela-Scafati O. (2002). Chemical composition of shallot (Allium ascalonicum Hort.). *Journal of Agricultural and Food Chemistry*.

[38] Taherkhani A., Orangi A., Moradkhani S., Jalalvand A., Khamverdi Z. (2022). Identification of potential anti-tooth-decay compounds from organic cinnamic acid derivatives by inhibiting matrix metalloproteinase-8: an in silico study. *Avicenna Journal of Dental Research*.

[39] Sova M. (2012). Antioxidant and antimicrobial activities of cinnamic acid derivatives. *Mini Reviews in Medicinal Chemistry*.

[40] de Cássia da Silveira e Sá R., Andrade L., dos Reis Barreto de Oliveira R., de Sousa D. P. (2014). A review on anti-inflammatory activity of phenylpropanoids found in essential oils. *Molecules*.

[41] Anantharaju P. G., Gowda P. C., Vimalambike M. G., Madhunapantula S. V. (2016). An overview on the role of dietary phenolics for the treatment of cancers. *Nutrition Journal*.

[42] Liu P., Hu Y., Guo D.-H. (2010). Potential antidepressant properties of radix polygalae (yuan zhi). *Phytomedicine*.

[43] Alam M. A., Subhan N., Hossain H. (2016). Hydroxycinnamic acid derivatives: a potential class of natural compounds for the management of lipid metabolism and obesity. *Nutrition & Metabolism*.

[44] Szwajgier D., Borowiec K., Pustelniak K. (2017). The neuroprotective effects of phenolic acids: molecular mechanism of action. *Nutrients*.

[45] Taherkhani A., Moradkhani S., Orangi A., Jalalvand A. (2021). In silico study of some natural anthraquinones on matrix metalloproteinase inhibition. *Research Journal of Pharmacognosy*.

[46] Xin D., Li H., Zhou S., Zhong H., Pu W. (2022). Effects of anthraquinones on immune responses and inflammatory diseases. *Molecules*.

[47] Naghibi F., Khalaj A., Mosaddegh M., Malekmohamadi M., Hamzeloo-Moghadam M. (2014). Cytotoxic activity evaluation of some medicinal plants, selected from Iranian traditional medicine Pharmacopoeia to treat cancer and related disorders. *Journal of Ethnopharmacology*.

[48] Abu N., Zamberi N. R., Yeap S. K. (2018). Subchronic toxicity, immunoregulation and anti-breast tumor effect of Nordamnacantal, an anthraquinone extracted from the stems of *Morinda citrifolia* L. *BMC Complementary and Alternative Medicine*.

[49] Cetin A. (2022). Some flavolignans as potent sars-cov-2 inhibitors via molecular docking, molecular dynamic simulations and ADME analysis. *Current Computer-Aided Drug Design*.

[50] Cetin A. (2021). In silico studies on stilbenolignan analogues as SARS-CoV-2 Mpro inhibitors. *Chemical Physics Letters*.

[51] Sureja D. K., Shah A. P., Gajjar N. D., Jadeja S. B., Bodiwala K. B., Dhameliya T. M. (2022). In silico computational investigations of AntiViral lignan derivatives as potent inhibitors of SARS CoV2. *ChemistrySelect*.

[52] Shah A., Patel V., Parmar B. (2021). Discovery of some antiviral natural products to fight against novel coronavirus (SARS-CoV-2) using an in silico approach. *Combinatorial Chemistry & High Throughput Screening*.

[53] Manoochehri H., Jalali A., Tanzadehpanah H., Taherkhani A., Najafi R. (2022). Aptamer-conjugated nanoliposomes containing COL1A1 siRNA sensitize CRC cells to conventional chemotherapeutic drugs. *Colloids and Surfaces B: Biointerfaces*.

[54] Lucet I. S., Fantino E., Styles M. (2006). The structural basis of Janus kinase 2 inhibition by a potent and specific pan-Janus kinase inhibitor. *Blood*.

[55] Burley S. K., Berman H. M., Bhikadiya C. (2019). RCSB Protein Data Bank: biological macromolecular structures enabling research and education in fundamental biology, biomedicine, biotechnology and energy. *Nucleic Acids Research*.

[56] Wishart D. S., Feunang Y. D., Guo A. C. (2018). DrugBank 5.0: a major update to the DrugBank database for 2018. *Nucleic Acids Research*.

[57] Masumi M., Noormohammadi F., Kianisaba F., Nouri F., Taheri M., Taherkhani A. (2022). Methicillin-resistant *Staphylococcus aureus*: docking-based virtual screening and molecular dynamics simulations to identify potential penicillin-binding protein 2a inhibitors from natural flavonoids. *International journal of microbiology*.

[58] Dinakarkumar Y., Rajabathar J. R., Arokiyaraj S. (2021). Anti-methanogenic effect of phytochemicals on methyl-coenzyme M reductase—potential: in silico and molecular docking studies for environmental protection. *Micromachines*.

[59] Goodsell D. S., Morris G. M., Olson A. J. (1996). Automated docking of flexible ligands: applications of AutoDock. *Journal of Molecular Recognition*.

[60] Racine J. (2000). *The Cygwin Tools: A GNU Toolkit for Windows*.

[61] Zhu K., Borrelli K. W., Greenwood J. R. (2014). Docking covalent inhibitors: a parameter free approach to pose prediction and scoring. *Journal of Chemical Information and Modeling*.

[62] Friesner R. A., Murphy R. B., Repasky M. P. (2006). Extra precision glide: docking and scoring incorporating a model of hydrophobic enclosure for protein− ligand complexes. *Journal of Medicinal Chemistry*.

[63] Azadian Z., Hosseini S., Dizjikan Z. P. (2021). Computational and in vitro validation of cardiogenic induction of quercetin on adipose‐derived mesenchymal stromal cells through the inhibition of Wnt and non‐Smad‐dependent TGF *β* pathways. *Journal of Cellular Biochemistry*.

[64] Brooks B. R., Brooks C. L., Mackerell A. D. (2009). CHARMM: the biomolecular simulation program. *Journal of Computational Chemistry*.

[65] Daina A., Michielin O., Zoete V. (2017). SwissADME: a free web tool to evaluate pharmacokinetics, drug-likeness and medicinal chemistry friendliness of small molecules. *Scientific Reports*.

[66] Parmar G., Shah A., Shah S., Seth A. K. (2022). Identification of bioactive phytoconstituents from the plant Euphorbia hirta as potential inhibitor of SARS-CoV-2: an in-silico approach. *Biointerface Res Appl Chem.*.

[67] de Sá A. G., Long Y., Portelli S., Pires D. E., Ascher D. B. (2022). toxCSM: comprehensive prediction of small molecule toxicity profiles. *Briefings in Bioinformatics*.

[68] Shah A. P., Parmar G. R., Sailor G. U., Seth A. K. (2019). Antimalarial phytochemicals identification from Euphorbia hirta against plasmepsin protease: an in silico approach. *Folia Medica*.

[69] Daina A., Zoete V. (2016). A boiled egg to predict gastrointestinal absorption and brain penetration of small molecules. *ChemMedChem*.

[70] Taherkhani A., Moradkhani S., Orangi A., Jalalvand A., Khamverdi Z. (2021). Molecular docking study of flavonoid compounds for possible matrix metalloproteinase-13 inhibition. *Journal of Basic and Clinical Physiology and Pharmacology*.

[71] Khamverdi Z., Mohamadi Z., Taherkhani A. (2022). Molecular docking and dynamics simulation of natural phenolic compounds with GSK-3*β*: a putative target to combat mortality in patients with COVID-19. *Recent advances in inflammation & allergy drug discovery*.

[72] Huang B., Lang X., Li X. (2022). The role of IL-6/JAK2/STAT3 signaling pathway in cancers. *Frontiers Oncology*.

[73] Jing S. Q., Yan L. J., Wang S. S. (2020). Neuroprotection of Cyperus esculentus L. orientin against cerebral ischemia/reperfusion induced brain injury. *Neural regeneration research*.

[74] An F., Wang S., Tian Q., Zhu D. (2015). Effects of orientin and vitexin from Trollius chinensis on the growth and apoptosis of esophageal cancer EC-109 cells. *Oncology Letters*.

[75] Kim S. J., Pham T. H., Bak Y., Ryu H. W., Oh S. R., Yoon D. Y. (2018). Orientin inhibits invasion by suppressing MMP-9 and IL-8 expression via the PKC*α*/ERK/AP-1/STAT3-mediated signaling pathways in TPA-treated MCF-7 breast cancer cells. *Phytomedicine*.

[76] Sharma P., Prakash O., Shukla A. (2016). Structure-activity relationship studies on holy basil (ocimum sanctum L.) based flavonoid orientin and its analogue for cytotoxic activity in liver cancer cell line HepG2. *Combinatorial Chemistry & High Throughput Screening*.

[77] Shafiq N., Zareen G., Arshad U. (2024). A mini review on the chemical and bio-medicinal aspects along with energy storage applications of anthraquinone and its analogues. *Mini-Reviews in Organic Chemistry*.

[78] Han N. R., Kim H. Y., Kang S. (2019). Chrysophanol, an anthraquinone from AST2017-01, possesses the anti-proliferative effect through increasing p53 protein levels in human mast cells. *Inflammation Research*.

[79] Rim H. K., Moon P. D., Choi I. H., Lee E. H., Kim H. M., Jeong H. J. (2013). SoSoSo or its active ingredient chrysophanol regulates production of inflammatory cytokines & adipokine in both macrophages & adipocytes. *Indian Journal of Medical Research*.

[80] Hayakawa S., Ohishi T., Miyoshi N., Oishi Y., Nakamura Y., Isemura M. (2020). Anti-cancer effects of green tea epigallocatchin-3-gallate and coffee chlorogenic acid. *Molecules*.

[81] Ranjbary A. G., Bagherzadeh A., Sabbaghi S. S. (2023). Chlorogenic acid induces apoptosis and cell-cycle arrest in colorectal cancer cells. *Molecular Biology Reports*.

[82] Sabanayagam R., Krishnamoorthy S., Anbuselvam M., Muruganantham B., Muthusami S. (2023). A comparative analysis of phyto-components on EGFR binding, viability, and migration in HPV positive ME180 and HPV negative C33A cervical cancer cells. *Medical Oncology*.

[83] Hsu P.-H., Chen W.-H., Juan-Lu C. (2021). Hesperidin and chlorogenic acid synergistically inhibit the growth of breast cancer cells via estrogen receptor/mitochondrial pathway. *Life*.

[84] Yuan C., Chen G., Jing C. (2022). Eriocitrin, a dietary flavonoid suppressed cell proliferation, induced apoptosis through modulation of JAK2/STAT3 and JNK/p38 MAPKs signaling pathway in MCF-7 cells. *Journal of Biochemical and Molecular Toxicology*.

[85] Li Y., Guo F., Chen T., Zhang L., Qin Y. (2021). Anthraquinone derivative C10 inhibits proliferation and cell cycle progression in colon cancer cells via the Jak2/Stat3 signaling pathway. *Toxicology and Applied Pharmacology*.

[86] Kim S. H., Park S. Y., Park Y. L., Myung D. S., Rew J. S., Joo Y. E. (2017). Chlorogenic acid suppresses lipopolysaccharide-induced nitric oxide and interleukin-1*β* expression by inhibiting JAK2/STAT3 activation in RAW264.7 cells. *Molecular Medicine Reports*.

[87] Tariq A., Mussarat S., Adnan M. (2015). Review on ethnomedicinal, phytochemical and pharmacological evidence of Himalayan anticancer plants. *Journal of Ethnopharmacology*.

[88] Gupta D. D., Mishra S., Verma S. S. (2021). Evaluation of antioxidant, anti-inflammatory and anticancer activities of diosgenin enriched Paris polyphylla rhizome extract of Indian Himalayan landraces. *Journal of Ethnopharmacology*.

[89] Manhas R. S., Ahmad S. M., Mir K. B. (2022). Isolation and anticancer activity evaluation of rare Bisaryl anthraquinone antibiotics from novel Streptomyces sp. strain of NW Himalayan region. *Chemico-Biological Interactions*.

